# Report of the Diseases of Edinburgh for November, 1808

**Published:** 1809-01

**Authors:** John Roberton

**Affiliations:** Surgeon


### *Report of the Diseases of Edinburgh for November,* 1808.
By John Roberton, Surgeon.

The weather during this month has been, in general, cloudy, with occasional showers of rain. From time to time, however, it was clear for one or two days, during which, particularly in the mornings, the frost was pretty evere. The easterly winds prevailed at different times during this month, when, as usual, the fogs occasioned by hem, though not to such an extent as we have frequently on former occasions observed, always accompanied them.

The barometer, during the whole month, was in general low, not varying, in any remarkable degree, by the changes of weather which occurred with us. The thermometer was generally about 40, except during the frost, when of course it was much lower, or during the clear days, when it sometimes stood as high as 50, or even 56.

Since the commencement of this month, chronic complaints have been very prevalent in various parts of the city and suburbs, such as rheumatism, asthma, &c. In many instances, they have proved extremely obstinate, particularly during the fogs; indeed, in some cases, I have been scarcely able to procure even partial relief till the easterly winds shifted, when they became more manageable.

Cynanche tonsillaris, with or without catarrh, has been very prevalent during the course of this month; and, although very obstinate, yet, by employing vigorous cathartic medicines, gargles, leeches, and, in many instances, a blister over the whole fore-part of the throat, the inflammation was, in general, prevented from proceeding to suppuration. In some cases, however, all these applications had no effect in preventing suppuration, and, in one or two, the tumor broke externally. The catarrhal symptoms, appearing independently of cynanche tonsillaris, were frequently accompanied with great tenderness of the eyes. Cathartic medicines relieved the first, while the application of a piece of loaf bread, dipt in cold water and applied over the eyes, never failed in curing the other. On account, however, of the particular state of the atmosphere, relapses were very common; it was therefore found necessary for such persons, as had recently suffered from this complaint, to expose themselves to the external air with the greatest caution.

While many cases of pneumonia are of a mild nature, several have occurred in practice of the greatest severity.

In

In some of these, indeed, the strictest perseverance in every plan of cure at present in use, did not yield that relief which might have been expected, as several of these cases terminated fatally, even after venesection, blistering, &c. had been repeatedly employed to the greatest extent which the system was capable of supporting.

When this disease, which is the case in several instances at present under my care, attacks those of a debilitated habit, and in whom, from various circumstances, it is impossible to employ blood-letting, there is perhaps no predicament in which a surgeon can be so unpleasantly situated. I have, however, by the greatest attention with the administration of saline purgatives and the repeated application of blisters to the breast, succeeded in removing the pneumonic symptoms. I have at present, two such patients, who, since the entire removal of pneumonia, have been affected with dropsical symptoms; and, from the state to which their former complaints had reduced them, with their previous vitiated habit in general, will probably, notwithstanding the strictest attention on my part, soon send them "to that undiscovered country from whose bourne no traveller returns."

Among infants, that inflammatory action of the system, which, unless actively treated is never checked, and, unless checked, always terminates in hydrocephalus internus, has come under my observation in various instances.

When early observed, and when the exciting causes, such as teething, which very commonly occasions that state of the system, has been removed by freely scarifying the gums, I have seldom, if ever, seen any of these cases terminate fatally. But when this has been neglected, and when the bowels, which, in this state of the system, are almost always costive, are not relieved by purgative medicines, the case seldom, if ever, terminates favorably. Under these circumstances, medical men, *never at a loss for accounting for every thing relating to disease*, console themselves by attempting to persuade the parents that their infant had died of what is generally known among them by the absurd names of *spaining brash, bowel hive, draughts, milk-fever, convulsions*, &c.

A blister, however, being applied over the whole head, known by the name of a *Cap Blister*, with the administration of brisk purgatives, the child seldom if ever fails in obtaining a complete recovery in a few days. In some cases, I have found it necessary, in addition to the blister,

&c. to apply leeches to the temples, and even to open the jugular vien.

I may remark, that in this, as well as in every other disease, I have uniformly observed that in families where scrofula prevails, I have never been so decidedly successful in my treatment, as in others where the existence of such a disease could not be traced.

Several cases of typhus fever, which perpetually prevails here, have lately been protracted to an amazing length of time. Two or three cases, notwithstanding the utmost attention that could be paid to them, have continued from thirty to forty days, and in some even longer. Neither of them have yet terminated fatally, but it is not to be expected, from the particular state of their system, that the patients will recover.

4, *St. James's Street.*

